# Antiresorptive treatments for corticosteroid-induced osteoporosis: a Bayesian network meta-analysis

**DOI:** 10.1093/bmb/ldac017

**Published:** 2022-06-01

**Authors:** Filippo Migliorini, Giorgia Colarossi, Jörg Eschweiler, Francesco Oliva, Arne Driessen, Nicola Maffulli

**Affiliations:** Department of Orthopaedic and Trauma Surgery, University Clinic Aachen, RWTH Aachen University Clinic, Aachen 52074, Germany; Department of Orthopaedic and Trauma Surgery, University Clinic Aachen, RWTH Aachen University Clinic, Aachen 52074, Germany; Department of Orthopaedic and Trauma Surgery, University Clinic Aachen, RWTH Aachen University Clinic, Aachen 52074, Germany; Department of Medicine, Surgery and Dentistry, University of Salerno, Via S. Allende, 84081 Baronissi (SA), Italy; Department of Orthopaedic and Trauma Surgery, University Clinic Aachen, RWTH Aachen University Clinic, Aachen 52074, Germany; Department of Medicine, Surgery and Dentistry, University of Salerno, Via S. Allende, 84081 Baronissi (SA), Italy; School of Pharmacy and Bioengineering, Keele University School of Medicine, Thornburrow Drive, Stoke on Trent, UK; Queen Mary University of London, Barts and the London School of Medicine and Dentistry, Centre for Sports and Exercise Medicine, Mile End Hospital, 275 Bancroft Road, London ST4 7JD, UK

**Keywords:** corticosteroid, osteoporosis, BMD, fracture, alendronate, drugs

## Abstract

**Introduction:**

Corticosteroid-induced osteoporosis (CIO) is the most common type of secondary osteoporosis, leading to fractures, and increased morbidity and mortality.

**Source of data:**

Pubmed, EMBASE, Scopus and Google Scholar databases.

**Areas of agreement:**

Prolonged glucocorticoids administration leads to secondary osteoporosis.

**Areas of controversy:**

The optimal management for CIO is controversial.

**Growing points:**

The present study compared bone mineral density, fractures and adverse events in patients undergoing treatment with risedronate, alendronate, zoledronate, denosumab or etidronate for CIO.

**Areas timely for developing research:**

For selected patients with CIO, alendronate performed better overall. These results must be interpreted within the limitations of the present study.

**Level of evidence:**

I, Bayesian network meta-analysis of randomized clinical trials.

## Introduction

Osteoporosis impacts negatively on bone turnover^1^: bone resorption exceeds formation, with progressive reduction of bone mass.^2^ The destruction of bone architecture and the rarefaction of bone mass, in turn, increase the rate of occurrence of pathological insufficiency fractures.^3^ Approximately 20% of Caucasian men and 50% of women older than 50 years sustain an osteoporotic fracture.^4^ In addition to gender, age and bone mineral density (BMD), other risk factors for pathologic fractures are history of prior fracture, parental history of hip fracture, consumption of tobacco or alcohol, rheumatoid arthritis and use of glucocorticoids.^5^ Fractures occur more frequently in the hip, spine and forearm,^6^ leading to a deterioration in quality of life, and increased morbidity and mortality.^7^

Prolonged glucocorticoids administration leads to secondary osteoporosis.^8^ Corticosteroids affect every tissue in the organism.^9^ High doses and/or long term administration of corticosteroids impacts osteocytes, through the overexpression of the macrophage colony-stimulating factor (M-CSF) and receptor activator of the nuclear factor kappa-B ligand (RANKL), and induces the reduction in osteoprotegerin, triggering rapid bone reabsorption.^10^ This progressive bone impairment is then exacerbated by a quantitative reduction of osteoblasts.^11^ Several pharmacological treatments have been proposed for corticosteroids-induced osteoporosis (CIO).^11^ For example, bisphosphonates (e.g. alendronate, risedronate, etidronate) are commonly used in selected patients.^12^^,^^13^ Other options for the management of CIO are teriparatide, a parathormone analogue promoting bone formation, and denosumab, an anti-resorptive agent.^14^^,^^15^ The best treatment for CIO, however, is still controversial.^15–18^ This Bayesian network meta-analysis compared BMD, fragility fractures, and adverse events in patients with CIO who underwent treatment with risedronate, alendronate, zoledronate, denosumab and/or etidronate.

## Material and methods

### Search strategy

This Bayesian network meta-analysis followed the PRISMA extension statement for reporting of systematic reviews incorporating network meta-analyses of health care interventions.^19^ The PICO framework was preliminary drafted:

- P (population): CIO;
- I (intervention): pharmacological therapy;
- C (comparison): risedronate, alendronate, zoledronate, denosumab, etidronate;
- O (outcomes): BMD, fragility fractures, and adverse events.

### Data source

Two authors (F.M. & G.C.) independently performed the literature search in April 2022. The search was firstly performed on Pubmed database. Subsequently, EMBASE, Scopus and Google Scholar databases were used to identify further articles. No time constraint was adopted for the literature search. The following keywords were used in this schema: osteoporosis [All Fields] AND glucocorticoid-induced [All Fields] COMBINED WITH bone loss [All Fields], BMD [All Fields], glucocorticoid [All Fields], steroids [All Fields], vertebral [All Fields], spine [All Fields], fracture [All Fields], hip [All Fields], femur [All Fields], bisphosphonate [All Fields], alendronate [All Fields], residronate, zoledronate [All Fields], denosumab [All Fields], PTH [All Fields], Teriparatide [All Fields], vitamin D [All Fields], calcium [All Fields]. The resulting publications were independently inspected by the same authors, and, if related to the topic at hand, they were considered for inclusion. The bibliographies of the full-text articles were also screened by hand to identify other articles eligible for inclusion.

### Eligibility criteria

All randomized clinical trials (RCTs) comparing treatments for CIO were considered. Given the authors’ language capabilities, articles in English, German, Italian, French and Spanish were eligible. Only peer reviewed RCTs of level I evidence, according to Oxford Centre of Evidence-Based Medicine,^20^ were considered. Studies analyzing the administration of only vitamin D and calcium were not included. Studies performed on patients with malignancy were not included. Clinical trials performed in children were also excluded. Studies performed on animals were not included. Only articles reporting quantitative data on at least one of the outcomes of interests were considered for inclusion.

### Study selection and data extraction

Two independent authors (F.M. & G.C.) performed data extraction. Generalities of the included RCTs were retrieved (author and years, journal, length of the follow-up). Patient baseline data were also extracted (calcium daily supplement (mg), vitamin D daily supplement (UI), type of treatment, route of administration, number of samples, mean age and percentage of women, BMD). In case of studies which several articles with different length of the follow-up were published, we collected demographic data for baseline assessment from the pivotal trial, while data at the latest follow-up were extracted for the network analyses. The following data at the latest follow-up were retrieved: (1) BMD of femoral neck, hip and spine, (2) fractures (non-spine, spine) and (3) adverse events (serious, and those leading to study discontinuation). The endpoint serious adverse events gather those necessitating hospital admission (e.g. abdominal pain, oesophagitis/gastritis, oesophageal/gastric/duodenal ulcers, severe hypo-hypercalcemia, severe infections, osteonecrosis, kidney failure).

### Methodological quality assessment

The methodological quality assessment was performed by two authors independently (F.M. & G.C.). The risk of bias summary tool of the Review Manager Software (The Nordic Cochrane Collaboration, Copenhagen) was used. The risk to incur in the following biases were evaluated: selection, detection, attrition, and other source of bias.

### Statistical analysis

The main author (F.M.) performed the statistical analyses. The STATA Software/MP version 16.1 (StataCorporation, College Station, Texas, USA) was used for statistical analyses. The Shapiro–Wilk test has been performed to investigate whether data have a normal distribution. Mean and standard deviation were evaluated for parametric data. The baseline comparability was assessed using analysis of variance (ANOVA), with *P* values > 0.1 considered satisfactory. Median and interquartile were evaluated for non-parametric data. The baseline comparability was assessed by the Kruskal–Wallis test, with *P* values > 0.1 considered satisfactory. A Bayesian hierarchical random-effects model analysis was used for the network comparisons. The inverse variance method was used for analysis. Continuous variables were analyzed using the standardized mean difference (SMD) effect measure, and the log odd ratio (LOR) was adopted for binary data. To obtain most reliable results, given the heterogeneous daily supplementation of calcium and vitamin D, the placebo group was not included in the network comparisons. Rather, all the variables were compared in the network analyses against a fictitious control group (no event). For continuous variables, the maximum score value was considered, while no event was considered for dichotomic outcomes. The overall inconsistency was evaluated through the equation for global linearity using the Wald test. If the *P* value was > 0.5, the null hypothesis could not be rejected, and the consistency assumption could be accepted at the overall level of each treatment. Both confidence (CI) and percentile (PrI) intervals were set at 95%. Edge plots were performed to display the number of direct comparisons, while interval plots were used to rank the treatment according to their effect size. Funnel plots were obtained to evaluate the risk of publication bias for each comparison.

## Results

### Search result

**Fig. 1:**
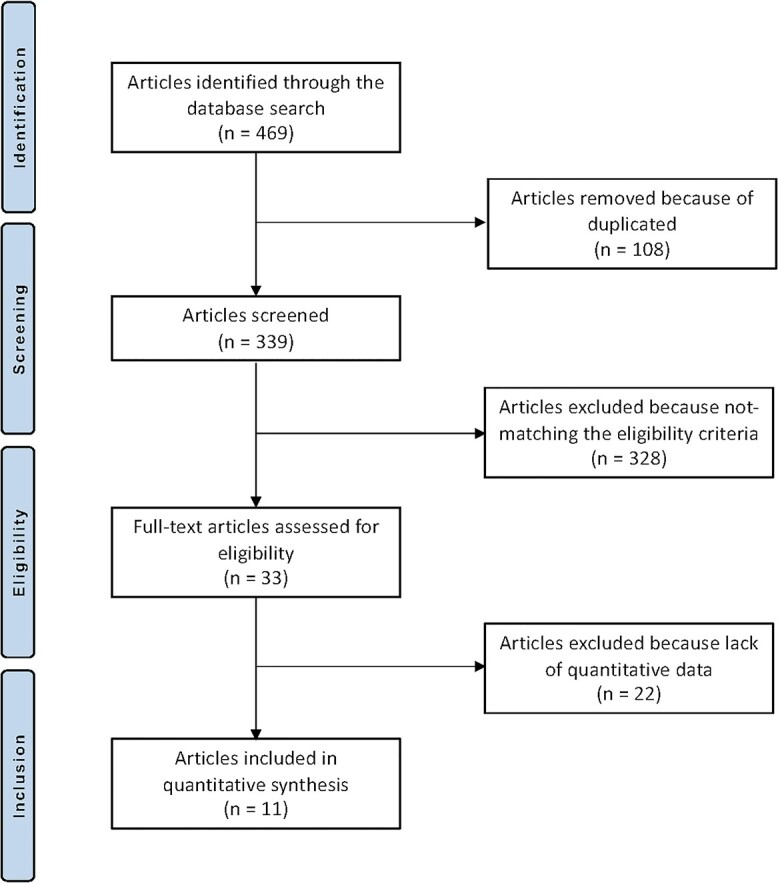
Flow chart of the literature search.

The literature search produced 447 articles, 108 of which were excluded because of duplication. An additional 328 articles were excluded because of: nature of the study (*N* = 77), non-clinical studies (*N* = 100), primary osteoporosis (*N* = 70), use of adjuvant(s) (*N* = 40), language limitations (*N* = 15) or uncertain results (*N* = 4). Another 22 articles were rejected because of missing quantitative data under the outcomes of interests. This left 11 RCTs for the present study. The literature search is shown in Figure 1.

### Methodological quality assessment

**Fig. 2:**
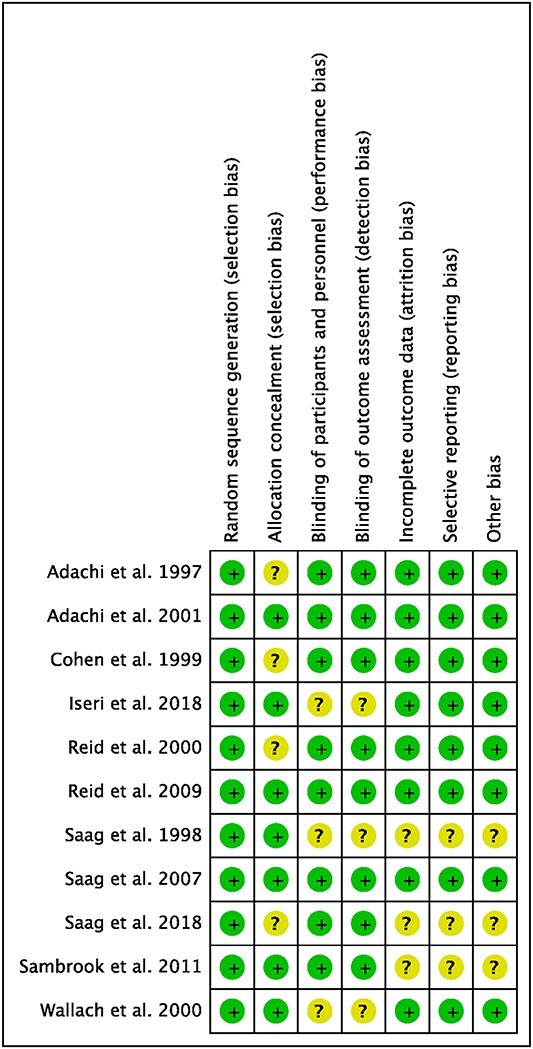
Methodological quality assessment.

The Cochrane bias of summary tool evidenced that all the included studies carried low risk of selection and detection biases, as only high-quality studies were included. Other biases (attrition, reporting, other) were also low. In conclusion, the quality of the methodological assessment was very good. The Cochrane bias of summary tool is shown in Figure 2.

### Patient demographics

Data from 4157 patients were collected. There were 2409/4157 (58%) women. The mean follow-up was 14.7 ± 4.9 months. The mean age was 58.4 ± 4.1 years. The mean BMD was 0.94 ± 0.06 g/cm^2^. The ANOVA test found comparability among the groups in terms of gender (*P* > 0.5), age (*P* > 0.5) and BMD (*P* > 0.5). Of note, in almost all included studies, patients were treated with additional daily supplements of calcium (mean 900.0 ± 223.6 mg) and vitamin D (mean 600.0 ± 230.9 units). Demographic data are reported in Table 1.

### Outcomes of interest

**Fig. 3:**
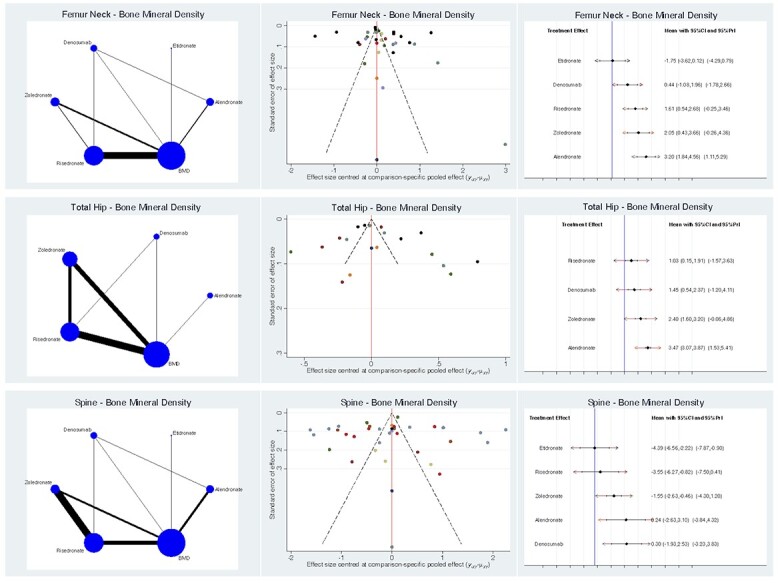
Overall results of the network comparisons concerning BMD.

At the end of the observation period, femoral neck BMD (SMD 3.2; 95% CI: 1.84–4.56) and hip BMD (SMD 3.5; 95% CI: 3.07–3.87) were greatest in the alendronate group (Figure 3). Concerning spine BMD (Figure 3), denosumab scored the highest (SMD 0.3; 95% CI: −1.93 to 2.53) followed closely by alendronate (SMD 0.2; 95% CI: −2.63 to 3.10). Regarding these comparisons, the test for global linearity resulted non-significant for inconsistency (*P* = 0.8, *P* = 0.8, *P* = 0.6, respectively).

**Fig. 4:**
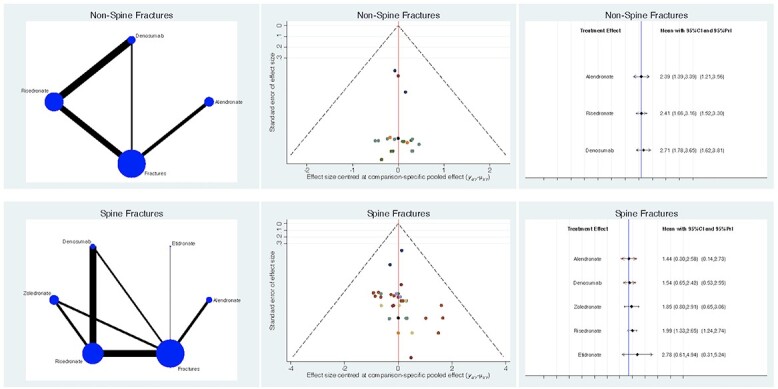
Overall results of the network comparisons concerning fractures.

Non-spine related fractures (Figure 4) were reduced in the alendronate group (LOR 2.4; 95% CI: 1.39–3.39). Spinal fractures (Figure 4) were also less common in the alendronate group (LOR 1.4; 95% CI: 0.30–2.58). Regarding these comparisons, the test for global linearity resulted non-significant for inconsistency (*P* = 0.7, *P* = 0.6, respectively).

**Fig. 5:**
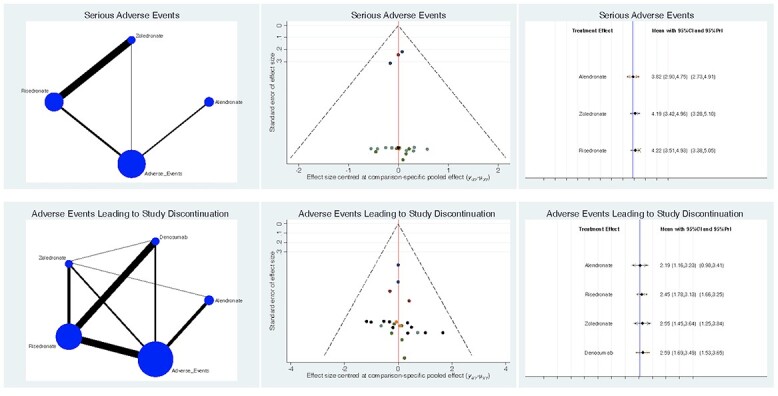
Overall results of the network comparisons concerning adverse events.

Serious adverse events were lowest in the alendronate group (LOR 3.8; 95% CI: 2.90–4.75). The alendronate group also reported the lowest rate of serious adverse events that required discontinuation of a study (LOR 2.2; 95% CI: 1.16–3.23). Regarding these comparisons, the test for global linearity resulted non-significant for inconsistency (*P* = 0.6, *P* = 0.8, *P* = 0.9, respectively). The overall results of the network comparisons concerning adverse events are shown in Figure 5.

## Discussion

The main findings of the present Bayesian network meta-analysis are that, for selected patients with CIO, alendronate performed better overall. Alendronate, risedonate, zoledronate and denosumab were all effective in increasing bone density in the spine and reducing vertebral fractures in patients taking corticosteroids; and alendronate, zoledronate and denosumab increased BMD in the hip. CIO is the most common form of secondary osteoporosis,^21^ and trabecular bone is normally more commonly affected.^22^ The intensity of bone rarefaction depends on the dose and duration of exposure to corticosteroids, with a dramatic increase in the risk of fractures in the first 3–6 months of treatment.^23^^,^^24^ The diagnosis is based on detecting changes in BMD through absorptiometry.^25^ The fracture risk assessment tool (FRAX), combined with clinical risk factors for osteoporosis with or without BMD measurements, is used to evaluate the potential risk of sustaining a major osteoporotic fracture in patients over 40 years old within 10 years.^26^

**Table 1:** Generalities and patient baseline data of the included studies

Author, year	Journal	Follow-up (months)	Calcium daily supplement (mg)	Vit D daily supplement (UI)	Type of treatment	Route of administration	Samples (*n*)	Mean age	Female (%)	BMD (spine)
Adachi et al. 2001 ^12^	*Arthritis & Rheumatism*	24	800–1000	250–500	Placebo	OS	61	54	69.00	0.93
					Alendronate	OS	53	53	71.00	0.92
					Alendronate	OS	55	53	73.00	0.93
					Alendronate	OS	29	56	52.00	0.89
Adachi et al. 1997 ^13^	*New England J Med*	12	500 mg/3x year		Etidronate	OS	67	62	61.00	0.94
					Placebo	OS	74	60	62.00	0.9
Cohen et al.1999 ^32^	*Arthritis & Rheumatism*	12	500		Risedronate	OS	75	60	66.70	1.032
					Risedronate	OS	76	62	64.50	1.082
					Placebo	OS	77	57	67.50	1.066
Iseri et al.2018 ^16^	*PLOS ONE*	12			Denosumab	SC	14	67	43.00	0.89
					Alendronate	OS	14	66	43.00	0.875
Reid et al.2009 ^17^	*The Lancet*	12			Zoledronate	IV	272	53	68.00	0.904
					Zoledronate	IV	144	56	69.00	0.902
					Risedronate	OS	273	53	67.00	0.898
					Risedronate	OS	144	58	69.00	0.958
Reid et al.2000 ^33^	*J Bone Mineral Res*	12	1000	400	Risedronate	OS	94	59	61.00	0.96
					Risedronate	OS	100	58	64.00	0.94
					Placebo	OS	96	59	62.00	0.93
Saag et al. 1998 ^31^	*New England J Med*	12	800–1000	250–500	Alendronate	OS	161	56	72.00	0.92
					Alendronate	OS	157	55	72.00	0.93
					Placebo	OS	159	54	67.00	0.95
Saag et al. 2007 ^14^	*New England J Med*	18	1000	800	Control	SC	214	56	80.40	0.85
					Alendronate	OS	214	57	80.80	0.85
Saag et al. 2018 ^15^	*The Lancet*	24	1000	800	Denosumab	SC	253	62	73.00	
					Denosumab	SC	145	68	64.00	
					Risedronate	OS	252	61	73.00	
					Risedronate	OS	145	64	64.00	
Sambrook et al.2011^18^	*Bone*	12	1000	400–1200	Zoledronate	IV	75	56	0.00	0.929
					Zoledronate	IV	38	59	0.00	1.004
					Risedronate	OS	77	53	0.00	0.92
					Risedronate	OS	40	63	0.00	1.026
Wallach et al.2000 ^30^	*Calcif Tissue Int*	12	500–1000	400	Risedronate	OS	165	59	63.00	0.991
					Risedronate	OS	174	59	64.00	1.003
					Placebo	OS	170	58	65.00	0.989

In selected patients, the use of chronic corticosteroids, as a targeted therapy, is necessary. Calcium and vitamin D supplements can be used to assist in the primary prevention of bone loss in patients using corticosteroids.^27^ However, the addition of bisphosphonates is effective in the management of primary and secondary osteoporosis as well.^28^^,^^29^ The administration of oral alendronate in patients with corticosteroid-induced osteoporosis produced significant improvement in BMD,^12^ which was associated with a statistically significant decrease in the risk of new vertebral fractures over 2 years of follow-up compared to placebo. The efficacy of increasing BMD using alendronate has been confirmed by the present network meta-analysis: alendronate resulted in statistically significant and clinically relevant better outcomes in femoral neck and hip BMDs compared to the other treatments analyzed. Other bisphosphonates such residronate have demonstrated effective reduction in the risk of new vertebral fractures by 70% and an increase in BMD in the lumbar spine, hip and radius.^30^ However, a RCT^17^ comparing oral administration of 5 mg of risedronate and intravenous infusion of 5 mg of zoledronate demonstrated the superiority of the latter in increasing BMD in the lumbar spine, trochanter, femoral neck and the whole hip area. The zoledronate group developed more adverse events in the first 3 days after infusion compared with the risedronate group. Interestingly, intermittent therapy with etidronate^13^ in patients using corticosteroids reduced the risk of new vertebral fractures by 40%. Additionally, anabolic drugs, such as teriparatide, also resulted in an increase in BMD and a reduction in the risk of fractures, compared with traditional anti-resorption drugs.^10^ Concerning 428 patients receiving teriparatide or alendronate,^14^ teriparatide was associated with a lower incidence of new vertebral fractures at 18 months (1:10). Non-vertebral fractures, however, were less common, but not significantly so, in the alendronate group. The present network meta-analysis demonstrated that alendronate was associated with a lower incidence of new vertebral fractures, in addition to non-vertebral fractures. A RCT comparing alendronate with denosumab identified a greater increase in lumbar spine BDM in the denosumab group.^16^ However, adverse events such as infections were more frequent in patients treated with denosumab compared to those receiving bisphosphonates. Another study^15^ comparing denosumab and risedronate reported no significant differences in the incidence of adverse events, including infections, between the groups. The present network meta-analysis confirmed the greater increase in lumbar spine BMD with denosumab, compared with the other drugs investigated, and also highlighted the higher rate of adverse events, particularly those leading to study discontinuation. In a RCT^31^ comparing alendronate versus placebo, serious adverse events or adverse events leading to drug discontinuation were not significantly different. Given the lack of data, etidronate was not considered in the other comparisons regarding adverse events. Alendronate showed better outcomes when focusing on serious adverse events, specifically those leading to study discontinuation.

This study presents some limitations. Given the lack of data in the current literature, indications for corticosteroids administration and duration of therapy were not considered. Likewise, differences in dosages were not analyzed. Data on daily vitamin D and calcium supplementation were often biased and most authors did not report the exact administration protocol; therefore, the impact of daily supplementation of calcium and vitamin D was not investigated. While the choice of the most effective dosage of medications used to prevent and treat osteoporosis remains controversial, in the present study different dose regimens were not compared, and this should be the objective of futures studies. An additional limitation of the present study is that clinical and morphometric forms of osteoporosis were not investigated separately. Biochemical markers of bone turnover were also not included in the analyses. Most of the included studies reported the results without considering the gender of the patients, and without reporting whether female patients were pre- or post-menopausal. There were undoubtedly differences among studies in the amount of time patients had been taking corticosteroids before starting an antiresorptive, and it is known that the response is greatest if treatment is started within about 3 months of the initiation of corticosteroids. Given the lack of quantitative data under the outcomes of interests, it was not possible to include teriparatide for analysis. Lack of information about the dose of steroids and underlying diseases requiring corticosteroids in the various studies may have also influenced the results of the present work. Baseline characteristics and fracture risks may well be different between the studies. These differences are not reported in most of the articles included, and may represent another weakness. According to our results, alendronate scored better overall. Only in the comparison of vertebral BMD denosumab scored better than alendronate. This difference is minimal, and therefore of dubious clinical relevance. However, we must underline that most CI are overlapping; thus, even if the level of inconsistency was acceptable, alendronate should not be used as default. Every patient must be individually framed and evaluated, and the treatment must be individualized according to their necessities. Therefore, given these limitations, data from the present Bayesian network meta-analysis must be interpreted in the lights of these limitations. Three protocols for clinical trials investigating the role of pamidronate, teriparatide, alendronate and daily supplement of dietary supplement of genistein aglycone have been currently registered, and investigations are ongoing (NCT03040531, NCT02472782, NCT00022841). Further studies are required to overcome current limitations and to reach more solid indications according to patient individualities and the underlying pathology peculiarities. The findings of the present investigation are derived from the results of randomized controlled clinical trials. They carry not only the advantages of RCT sourced data but also the limitations inherent in extrapolating them to routine clinical practice. The performance of drugs in RCTs, which are often of limited duration and involve limited numbers of selected patients, can differ significantly from their performance in routine practice. This is particularly relevant with essentially non-symptomatic diseases such as osteoporosis, where non adherence can be a major factor. Potentially important treatment complications can also fail to show up within the confines of a RCT. The concern about accelerated bone loss and a possible increase in vertebral fractures with cessation of denosumab therapy is such an example.

## Conclusion

Alendronate, risedonate, zoledronate and denosumab are all effective in increasing bone density in the spine and reducing vertebral fractures in patients taking corticosteroids. Alendronate, zoledronate and denosumab increased BMD in the hip. Alendronate produced increased femoral neck and hip BMDs, reduced incidence of novel fractures, and lower incidence of serious adverse events, specifically those leading to study discontinuation. These results must be interpreted within the limitations of the present study.

## Conflict of interest statement

The authors declare that they have no conflicts of interest.

## Funding

No external source of funding was used.

## Ethical approval

This article does not contain any studies with human participants or animals performed by any of the authors.

## Informed consent

For this type of study informed consent is not required.

## Data availability

The data underlying this article are available in the article and in its online supplementary material.
